# Dysphagia and disrupted cranial nerve development in a mouse model of DiGeorge (22q11) deletion syndrome

**DOI:** 10.1242/dmm.012484

**Published:** 2013-12-19

**Authors:** Beverly A. Karpinski, Thomas M. Maynard, Matthew S. Fralish, Samer Nuwayhid, Irene E. Zohn, Sally A. Moody, Anthony-S. LaMantia

**Affiliations:** 1 Department of Anatomy and Regenerative Biology, The George Washington University School of Medicine and Health Sciences, Washington DC 20037, USA.; 2 Department of Pharmacology and Physiology, The George Washington University School of Medicine and Health Sciences, Washington DC 20037, USA.; 3 The George Washington Institute for Neuroscience, Center for Neuroscience Research, Childrens National Medical Center, Washington DC 20010, USA.; 4 Department of Pediatrics, The George Washington University School of Medicine and Health Sciences, Washington DC 20037, USA.

**Keywords:** DiGeorge, 22q11 deletion syndrome, Cranial nerve development, Dysphagia, Hindbrain patterning

## Abstract

We assessed feeding-related developmental anomalies in the *LgDel* mouse model of chromosome 22q11 deletion syndrome (22q11DS), a common developmental disorder that frequently includes perinatal dysphagia – debilitating feeding, swallowing and nutrition difficulties from birth onward – within its phenotypic spectrum. *LgDel* pups gain significantly less weight during the first postnatal weeks, and have several signs of respiratory infections due to food aspiration. Most 22q11 genes are expressed in anlagen of craniofacial and brainstem regions critical for feeding and swallowing, and diminished expression in *LgDel* embryos apparently compromises development of these regions. Palate and jaw anomalies indicate divergent oro-facial morphogenesis. Altered expression and patterning of hindbrain transcriptional regulators, especially those related to retinoic acid (RA) signaling, prefigures these disruptions. Subsequently, gene expression, axon growth and sensory ganglion formation in the trigeminal (V), glossopharyngeal (IX) or vagus (X) cranial nerves (CNs) that innervate targets essential for feeding, swallowing and digestion are disrupted. Posterior CN IX and X ganglia anomalies primarily reflect diminished dosage of the 22q11DS candidate gene *Tbx1*. Genetic modification of RA signaling in *LgDel* embryos rescues the anterior CN V phenotype and returns expression levels or pattern of RA-sensitive genes to those in wild-type embryos. Thus, diminished 22q11 gene dosage, including but not limited to *Tbx1*, disrupts oro-facial and CN development by modifying RA-modulated anterior-posterior hindbrain differentiation. These disruptions likely contribute to dysphagia in infants and young children with 22q11DS.

## INTRODUCTION

Dysphagia – disrupted feeding, swallowing and nutrition – is a serious complication of several developmental disorders, including 22q11 deletion syndrome (22q11DS) (Christensen, 1989; Ang et al., 1999; Eicher et al., 2000; Schwarz et al., 2001; Rommel et al., 2008). Perinatal dysphagia is especially challenging to manage (Schwarz et al., 2001; Kelly, 2006; Lefton-Greif, 2008), and frequently results in aspiration-based infection and other complications (Hopkin et al., 2000; Trinick et al., 2012). Nevertheless, there is little appreciation of developmental pathogenic mechanisms that underlie dysphagia and, to our knowledge, no genetic models with early disruption of feeding and swallowing to facilitate detailed analysis. Thus, we investigated whether the *LgDel* mouse, a genomically accurate 22q11DS model that carries a heterozygous deletion of 28 contiguous genes on mouse chromosome 16 parallel to the minimal critical deleted region in 22q11DS patients (Merscher et al., 2001), provides a robust animal model for key features of dysphagia in 22q11DS and perhaps other neurodevelopmental disorders.

Dysphagia probably contributes to diminished weight gain (Tarquinio et al., 2012), increased naso-sinus and lung infections, and gastrointestinal reflux in 22q11DS infants and children. Some difficulties might reflect cardiovascular or immunological anomalies (Jawad et al., 2001); however, nasopharyngeal and airway dysmorphology (Huang and Shapiro, 2000; Marom et al., 2012) as well as altered oro-facial sensory and/or motor control (Zori et al., 1998) probably initiates or exacerbates aspiration or reflux that leads to discomfort and infection (Lundy et al., 1999; Rommel et al., 2008; Lima et al., 2010; Trinick et al., 2012). The pathogenesis of these complications remains unknown, presumably because developmental causes cannot be easily studied in 22q11DS patients. Thus, we assessed key signs of dysphagia in the *LgDel* mouse to determine whether feeding and swallowing is compromised perinatally, and whether developmental correlates of these changes could be studied in this animal model.

Optimal oro-facial morphogenesis is crucial for normal feeding and swallowing (Reilly et al., 1999; Barrow et al., 2000). Oro-facial development depends critically on appropriate levels and patterns of key regulatory genes first in the embryonic hindbrain and then in craniofacial primordia derived from hindbrain neural crest. Subsequent innervation by the trigeminal (V), facial (VII), glossopharyngeal (IX), vagus (X) and hypoglossal (XII) nerves is crucial for effective oro-facial sensory and/or motor control. There is currently no evaluation of disrupted oro-facial development, hindbrain gene expression or cranial nerve (CN) differentiation in the *LgDel* 22q11DS mouse model. Accordingly, we evaluated palate and jaw morphogenesis, expression of genes involved in hindbrain regionalization, CN differentiation and axon guidance, and identified phenotypes in CNs critical for feeding and swallowing in developing *LgDel* mice. In parallel, we investigated whether phenotypic changes reflect broad 22q11 deletion or that of the 22q11DS candidate gene *Tbx1*.

Weight gain, respiratory health and craniofacial morphogenesis are altered in *LgDel* mice. Gene expression levels and patterns change in the developing hindbrain and trigeminal ganglia. In parallel, hindbrain and CN differentiation is disrupted. Anterior CN phenotypes reflect broad 22q11 gene deletion and altered hindbrain RA signaling; posterior phenotypes reflect haploinsufficiency of the 22q11DS candidate gene *Tbx1* (Scambler, 2010). Thus, diminished 22q11 gene dosage in the *LgDel* model of 22q11DS, including – but not limited to – *Tbx1*, disrupts oro-facial development and function. Parallel changes in 22q11DS patients likely contribute to clinically significant feeding and swallowing difficulties during early life.

**TRANSLATIONAL IMPACT**

**Clinical issue**

Pediatric dysphagia – compromised food ingestion, chewing and swallowing – is a major complication for children with developmental disorders. Indeed, these difficulties are seen in up to 80% of children with developmental disorders. The consequences of pediatric dysphagia can be devastating and include diminished food intake, decreased weight gain, inadequate nutrition, choking, food aspiration, and subsequent naso-sinus, inner ear and respiratory infections including pneumonia. Despite these consequences, and their burden for the health and growth of children with developmental disorders, little is known about the etiology of pediatric dysphagia, in part because its pathology arises during fetal development and thus cannot be studied in patients, and in part because there are no animal models that encompass the key signs of the disease.

**Results**

In this study, the authors report that dysphagic symptoms, including diminished weight gain, nasopharyngeal milk aspiration, and naso-sinus, inner ear and lung infections, develop during early postnatal life in the *LgDel* mouse model of DiGeorge (22q11.2) deletion syndrome (22q11DS), a common developmental disorder with a substantial incidence of pediatric dysphagia. They show that these symptoms are prefigured by altered expression and patterning of genes in embryonic domains that generate the oro-pharyngeal structures and the cranial nerves critical for feeding and swallowing, and that cranial nerve development is disrupted in the *LgDel* mouse. These disruptions reflect contiguous gene effects within the 22q11-deleted region. Altered trigeminal nerve development is mediated by retinoic acid (RA)-sensitive genes, they report, and is rescued by diminished RA signaling in *LgDel* mice. Finally, the authors demonstrate that altered glossopharyngeal and vagal nerve development reflects *Tbx1* haploinsufficiency, which is also implicated in the cardiovascular phenotypes of 22q11DS.

**Implications and future directions**

These findings identify an animal model for pediatric dysphagia that will permit the detailed study of the craniofacial and nervous system developmental disruptions that cause the disorder, and of the peripheral and central nervous system circuitry that is compromised. The authors’ genetic rescue experiments provide a foundation for potential amelioration of pathogenesis in the animal model, and eventually in patients. The *LgDel* mouse can also be used as a resource to help identify genes and environmental exposures that exacerbate pediatric dysphagia. Finally, the *LgDel* mouse provides a tool for devising new ways to predict which patients will develop the challenging clinical complications of pediatric dysphagia and for devising therapies to ameliorate the symptoms of the condition.

## RESULTS

### Evidence of altered feeding and swallowing in *LgDel* mice

**Fig. 1.**
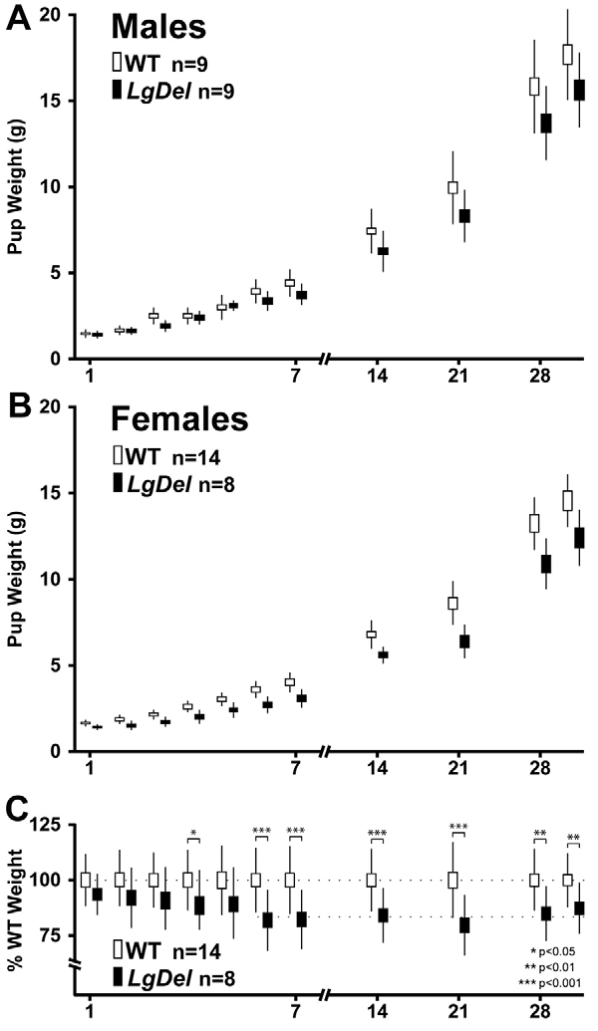
**Growth curves for *LgDel* and WT littermate males and females from P1 through P30.** (A) Growth curves for male *LgDel* and WT littermates, weighed daily from P1 through P7, then weekly from P7 through P28 (*x*-axis). (B) Growth curve for female *LgDel* and WT littermates. (C) Normalized mean weights for male and female *LgDel* and WT littermates. The boxes represent standard errors of each mean (s.e.m.), and bars reflect standard deviations for each data point. Growth curves were compared by ANOVA (A,B), and mean normalized values compared using Mann-Whitney analysis (C).

From birth onward, 22q11DS patients fail to gain weight at the same rates as typically developing children (Tarquinio et al., 2012), have difficulties ingesting and swallowing, and a high incidence of aspiration-related naso-sinus, respiratory and inner ear infections (Eicher et al., 2000; Hopkin et al., 2000; Jawad et al., 2001). To determine whether *LgDel* pups exhibit similar defining features of pediatric dysphagia, we first investigated whether they weigh the same as wild-type (WT) counterparts at birth, but fail to gain weight similar to WT littermates over the first postnatal month. *LgDel* pups of both sexes – analyzed separately – weigh the same as WT littermates at birth; however, *LgDel* weight gain slows from postnatal day 4 (P4) through P30 (*P*≤0.0004, two-way ANOVA; *n*=9 *LgDel*, 9 WT males; *P*≤0.0001, 8 *LgDel*, 14 WT females). To confirm the significance of these differences, independent of sex, we normalized *LgDel* weights to those of WT male or female counterparts (Fig. 1C). *LgDel* pups are first significantly lighter at P4 (although the P5 difference does not reach significance) through P30 (Mann-Whitney, *P*≤0.001; Fig. 1C). By adolescence, *LgDel* weight is 80% (males) to 85% (females) of their WT counterparts, similar to the 15% decrease in body weight of adolescent 22q11DS patients versus typically developing controls (Tarquinio et al., 2012).

**Fig. 2.**
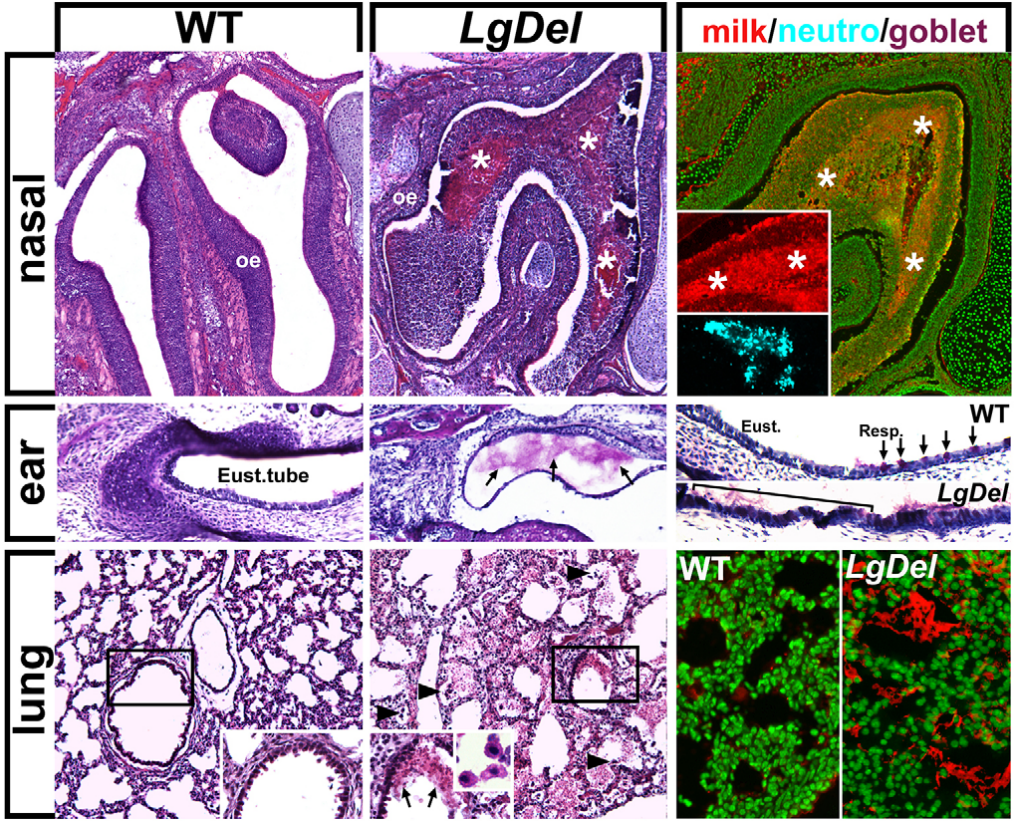
**P7 *LgDel* mice show signs of dysphagia, including nasal, ear and lung milk-aspiration inflammation or infection.** Top row: aspiration-related protein aggregates adjacent to the turbinates of the olfactory epithelium (oe) in P7 WT and *LgDel* pups. The protein aggregates (asterisks), seen only in *LgDel* pups, contain murine milk protein (far right; fluorescent Nissl stain, green; immunolabel for milk protein, red) as well as neutrophils (anti-CD64, blue). Middle row: mucus aggregates are seen in the Eustachian tubes of *LgDel* P7 pups, accompanied by an apparent increase in the frequency of mucus-producing goblet cells (bracket/arrows, far right panel; PAS stain) at the boundary of the pharynx (Resp.; arrows) and Eustachian (Eust.; bracket) tube epithelium. Bottom row: *LgDel* lungs have more frequent evidence of inflammation and/or infection, including red blood cell aggregates (arrows, middle insert), macrophages (arrowheads, and inset, middle) and infiltration of murine milk protein (far right panel; green, fluorescent Nissl stain; red, milk protein immunolabel).

We next investigated whether milk was aspirated into respiratory passages or whether there were signs of aspiration-related irritation or infection in *LgDel* pups or WT littermates. In the nasal sinuses, Eustachian tubes and lungs of *LgDel* but not WT littermate P7 pups, we found protein-rich aggregates that included murine milk protein, bacteria, red blood cells, macrophages and neutrophils (Fig. 2; *P*≤0.05, chi square; *n*=*LgDel*: 4/5, WT: 1/4). In the nasal sinuses, there were large accumulations of murine milk protein, frequently infiltrated with CD-64-positive neutrophils (Fig. 2, top right). In the Eustachian tubes, we found mucus accumulations and an apparent increase in mucus-producing goblet cells (Fig. 2, middle row), which are signs of ear infection (Cayé-Thomasen and Tos, 2003). In the lungs, milk protein aggregates accompanied red blood cells and macrophages (Fig. 2, bottom row and inset), also signs of infection. Thus, increased milk aspiration, accumulation of red blood cells, mucus-producing cells and immune cells, together with diminished weight gain, show that *LgDel* pups have many features that parallel clinical signs of dysphagia in infants and children with 22q11DS.

### Craniofacial changes in *LgDel* mice

**Fig. 3.**
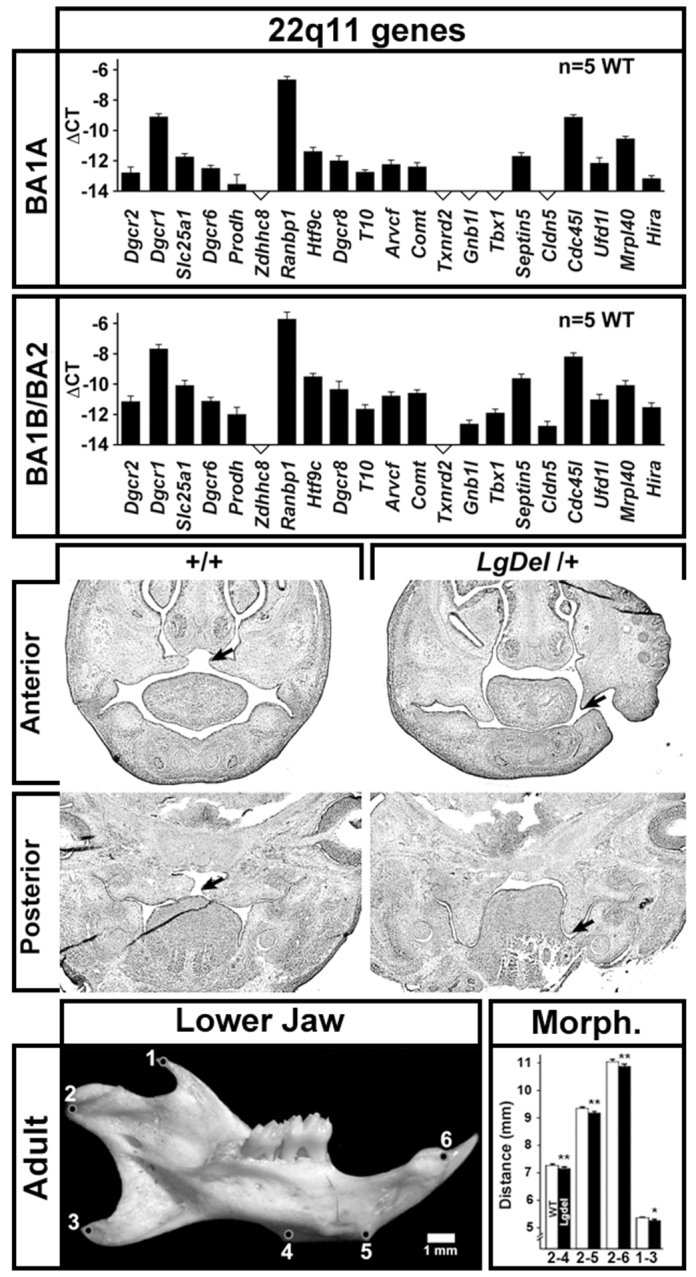
**Altered palate and jaw morphology in *LgDel* mice.** Top: quantitative real time PCR (qPCR) analysis of a subset of 22q11 deleted genes in microdissected rudiments of key orofacial structures for feeding and swallowing: BA1A, which includes the maxilla, which gives rise to the upper jaw, mouth structures and some muscles of mastication, and BA1B/BA2, which includes the mandible and hyoid, which gives rise to the lower jaw and some pharyngeal structures. These 22q11 genes were chosen based upon expression above a threshold level – 0.01% of *Gapdh* expression measured in the same sample – in a previous analysis of developing E10.5 embryos or the brain from E12.5 onward (Maynard et al., 2003). Expression was quantified. Middle: sections through the anterior and posterior palatal region in E13.5 WT and *LgDel* embryos demonstrating failure of palatal elevation in the *LgDel* (compare arrows). The palate has not fused in either the WT or *LgDel* at this age. WT palatal shelves are more frequently elevated (left arrows) than those in the *LgDel* (right arrows). Bottom left: lateral view of the mandibular process from a WT mouse, with reference points for measurements indicated as numbers 1–6. Bottom right: morphometry of point-to-point distances between cardinal locations shown at left indicates that mandibular growth is diminished in *LgDel* versus WT littermate mice (*P≤0.5; **P≤0.01, *t*-test; *n*=48 *LgDel*, 46 WT).

22q11DS patients have palate and jaw anomalies (Fukui et al., 2000; Emanuel et al., 2001; Ousley et al., 2007) that contribute to feeding and swallowing difficulties. It is not clear, however, whether 22q11 genes act in palate or jaw primordia, and whether diminished dosage alters morphogenesis in *LgDel* mice. We investigated whether a substantial number of 22q11 genes are expressed in embryonic structures that contribute to the palate, upper jaw and lower jaw (Fig. 3, top). We screened 21 candidates from the deleted region based on previous assessments of expression in the embryo and developing brain (Maynard et al., 2003; Meechan et al., 2009; Maynard et al., 2013). Using quantitative RT-PCR analysis (qPCR) in microdissected samples of maxillary process/branchial arch (BA1A; Fig. 3, top) or a combined mandibular/hyoid sample (BA1B/BA2; Fig. 3, middle top) consisting of the mandibular process (BA1B) and the hyoid process (BA2), we found 16/28 (BA1A) and 19/28 (BA1B/BA2) 22q11 genes expressed in the craniofacial primordia. Thus, local diminished dosage of 22q11 genes might compromise craniofacial morphogenesis.

The consequences of diminished 22q11 gene dosage in 22q11DS patients include, with variable penetrance, compromised palatal elevation that results in velopharyngeal insufficiency (Ruda et al., 2012). We therefore examined the developing palate in *LgDel* mice for signs of dysmorphogenesis, including failed elevation of the palatal shelves during fetal development as well as thinned palatal cartilage in the adult. In mid-gestation (E13.5) *LgDel* embryos, we found evidence of failed palatal shelf elevation (Fig. 3, middle), albeit at fairly low penetrance (2/7 *LgDel* versus 0/7 WT), parallel to low penetrance of similar phenotypes in 22q11DS patients (Kobrynski and Sullivan, 2007). We found no clear evidence of palatal cartilage thinning or other signs of palatal dysmorphogenesis in P7 *LgDel* pups (not shown), perhaps because severely affected pups are lost at or soon after birth. 22q11DS patients also have mandibular anomalies, including retrognathia (Friedman et al., 2011). In P30 *LgDel* mice, mandible size and shape are altered. Distances between the ventral condylar process, points on the masseteric ridge and the alveolus of the incisor (Fig. 3, bottom left) are decreased relative to WT (Fig. 3, bottom right; *P*≤0.005, *t*-test; *n*=*LgDel* 47, WT 46 hemi-mandibles). Thus, low-penetrance palate anomalies and mature mandible dysmorphology indicate that oro-facial development related to feeding and swallowing is altered in *LgDel* mice.

### Altered hindbrain morphogenesis, gene expression and patterning in *LgDel* embryos

**Fig. 4.**
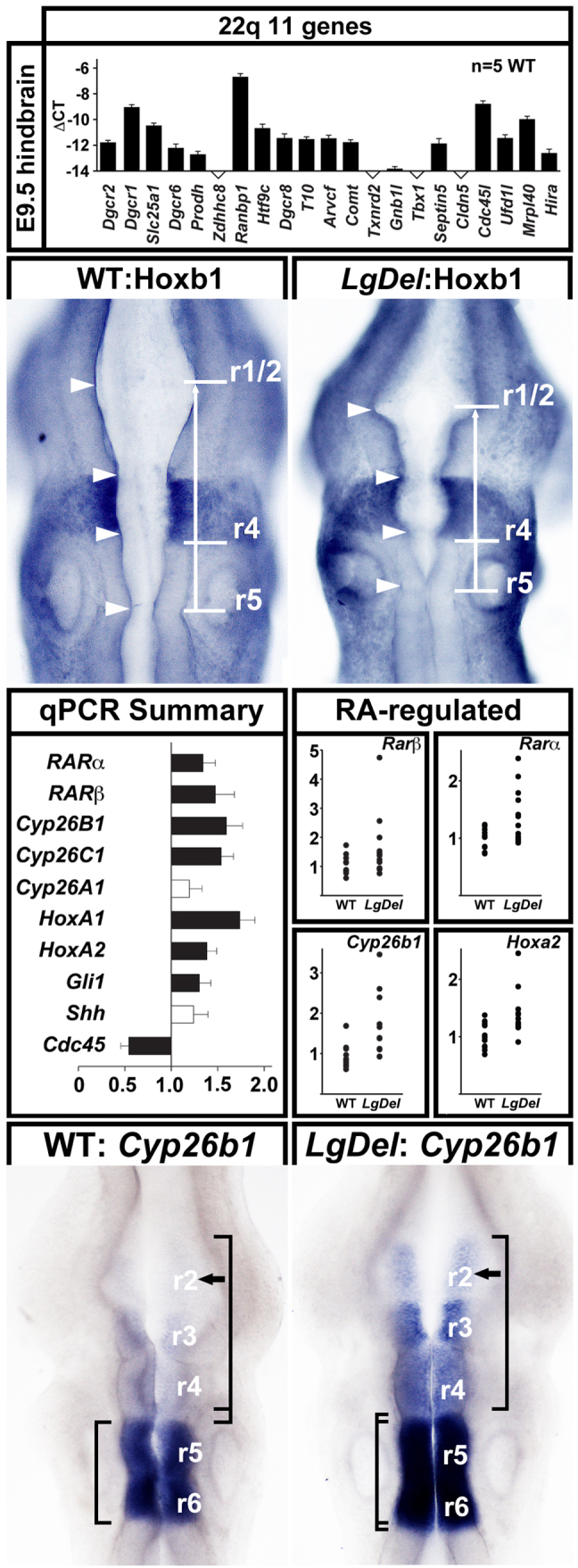
**Altered gene expression, morphogenesis and patterning in the *LgDel* E9.5 anterior hindbrain.** Top: qPCR analysis of 22q11 gene expression above threshold levels in the microdissected E9.5 hindbrain (r1 to r8). 16 out of 21 candidates are expressed above threshold. Note that *Tbx1*, a key 22q11 candidate gene for cardiovascular phenotypes, is not detected in the E9.5 hindbrain. Middle top: apparent pattern changes and dysmorphogenesis in the E9.5 anterior hindbrain. The distance between r4 and r1/2 in the WT, defined by Hoxb1 and En1 immunolabeling (left) is greater than that in the *LgDel* (right; compare white bars and arrow), and the dorsal margins of the neuroepithelium appear deformed (compare arrowheads). Middle bottom left: summary of qPCR analysis of expression of several genes that distinguish posterior (top) from anterior (bottom) rhombomeres in the E9.5 microdissected hindbrain (r1 to r8). Middle bottom right: scatterplots of expression levels in individual hindbrain samples of four RA-regulated genes – two found in posterior rhombomeres (*Rarα*, *Rarβ*), two with distinctly patterned expression in both anterior and posterior rhombomeres (*Cyp26b1*, *Hoxa2*). Bottom: (left) in the WT E9.5 hindbrain, *Cyp26b1* is expressed at high levels in r5/6, lower levels in r3/4 and is barely detectable in r2. (Right) In the *LgDel* E9.5 hindbrain from an embryo hybridized concomitantly with the WT at left, *Cyp26b1* is increased in r5/6, significantly stronger in r3/4 and is now clearly detectable in r2 (compare arrows, left and right). In addition, r5/6 boundaries have expanded in the *LgDel* (compare left brackets), and r2 to r4 have contracted (compare right brackets).

Optimal oro-facial development depends upon the emergence of rhomobomeres – metameric units that prefigure anterior-posterior (A-P) CN differentiation – and associated gene expression. To assess whether aberrant rhombomere morphogenesis and patterning prefigures *LgDel* dysphagia-related changes, we determined whether 22q11 genes could influence the developing hindbrain based upon local expression. In micro-dissected E9.5 hindbrain samples (rhombomere r1 to r8), we detected substantial expression of 17 out of 21 relevant 22q11 genes (Fig. 4, top). Four genes, including *Tbx1*, that are associated with cardiovascular and other 22q11DS phenotypes (Scambler, 2010) fell below confident detection levels (≤0.01% of *Gapdh* in the same sample). Thus, local diminished expression of a substantial subset of 22q11 genes could alter hindbrain morphogenesis, differentiation and patterning.

We assessed hindbrain differentiation by evaluating regions anterior and posterior to cardinal expression domains, defined by dual Hoxb1 and En1 immunolabeling. The E9.5 *LgDel* anterior hindbrain (Fig. 4, arrows, top middle) appeared compressed, and dorsal margins of the neuroepithelium appeared deformed (Fig. 4, arrowheads, top middle). Thus, anterior – but not posterior – rhombomeres seem relatively smaller and dysmorphic in the *LgDel* versus WT. We next investigated whether expression of a subset of retinoic acid (RA) co-factors and Hox genes, which, when dysregulated, disrupt A-P rhombomere identity (Marshall et al., 1992) (reviewed by Glover et al., 2006), was altered using qPCR in E9.5 microdissected hindbrains (r1–r8). *Rarα* and *Rarβ*, RA receptors found in posterior rhombomeres, increase by 34 and 47%, respectively (*P*≤0.04 and 0.02, Mann-Whitney, *n*=12 *LgDel*, 12 WT). *Cyp26b1*, an RA-regulated RA catabolic enzyme expressed at distinct levels in r6 to r2 (Abu-Abed et al., 1998; Reijntjes et al., 2003; Okano et al., 2012), is increased by 160% (*P*≤0.009), and *Cyp26c1* (r2) (Tahayato et al., 2003) is increased by 54% (*P*≤0.004). *Hoxa1* (r4–r8) increased by 74% (*P*≤0.001), and *Hoxa2* (r2–4 and posterior) by 38% (*P*≤0.04). The Shh transcriptional effector *Gli1* (r1) increases by 30% (*P*≤0.04). In contrast, *Shh* expression (no A-P distinction) was unchanged. We observed no significant differences for any of the RA-regulated genes in E9.5 *LgDel* versus WT cervical/thoracic spinal cord (data not shown); the changes we detect are limited to anterior or posterior hindbrain domains. Individual hindbrain expression levels varied; nevertheless, minimum and maximum levels were always higher in *LgDel* versus WT (Fig. 4, middle bottom right). Apparently, 22q11 deletion modifies gene expression levels that distinguish anterior or posterior rhombomeres, including several RA-regulated genes.

These expression level changes suggest that anterior rhombomere patterning is disrupted, potentially via posteriorizing influences of RA. Thus, we evaluated the expression pattern of *Cyp26b1*, which is known to be RA regulated (see above), has been implicated in determining RA-responsiveness of hindbrain cells (Hernandez et al., 2007) and whose altered expression – especially in the context of null mutations of the 22q11 candidate gene *Tbx1* (see below) – can compromise craniofacial development (Roberts et al., 2006; Okano et al., 2008; Okano et al., 2012). Bounded patterns and gradients of *Cyp26b1* distinguish posterior from anterior rhombomeres. In the WT hindbrain, there is intense expression in r5/6, progressively diminished expression in r3/4 and barely detectable expression in r2 (Fig. 4, bottom left panel). In the *LgDel* hindbrain, we found increased *Cyp26b1* signal in r6 through r3, and enhanced expression in r2, substantially above levels seen in any of the WT embryos (Fig. 4, bottom right panel; seen in 8/11 *LgDel* embryos versus 0/10 WT). These pattern changes, which parallel *Cyp26b1* level changes, are consistent with anomalous acquisition of posterior character in *LgDel* anterior rhombomeres, perhaps due to aberrant RA signaling.

### Disrupted CN and CNg development in *LgDel* embryos

**Fig. 5.**
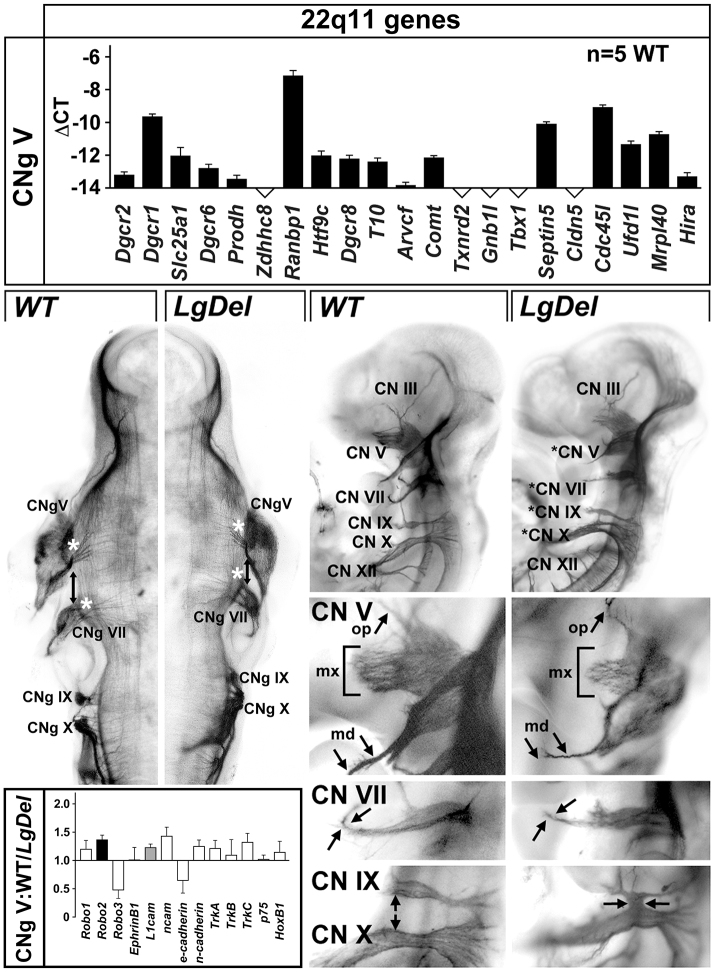
**CN development is altered in E10.5 *LgDel* embryos.** Top: 16 out of 21 22q11 deleted genes are expressed above threshold in CNg V, based upon qPCR analysis of microdissected WT E10.5 CNg V samples. Middle left: a representative E10.5 WT embryo labeled immunocytochemically for neurofilament protein shows the E10.5 WT hindbrain and associated cranial nerves (CN) and ganglia (CNg). In the *LgDel* (right), CNg V and VII appear more closely spaced (arrow), their motor roots less differentiated (asterisks), and, parallel to E9.5, the anterior hindbrain appears compressed. In addition, CNg IX and X appear fused in the LgDel (arrows). Bottom left panel: expression changes in *LgDel* CNg V determined by qPCR of microdissected ganglia from *LgDel* versus WT E10.5 embryos. *Robo2* (black bar) is significantly increased (*P*≤0.03) and L1cam (gray bar) shows a significant trend toward increased expression (*P*≤0.06). Middle right: (left) a lateral view of typical WT axon trajectories and fasciculation as well as the position of cranial sensory ganglia for a subset of CNs. (Right) CN development is altered in an E10.5 *LgDel* embryo (asterisks). CN V appears sparse and de-fasciculated, the normal bifurcation of CN VII is not evident, and the ganglia of CN IX and X are fused. Middle bottom right: specific CN phenotypes in *LgDel* embryos. These examples, from additional WT and *LgDel* embryos, are representative of the features scored for quantitative phenotypic analysis. First row: the ophthalmic (op, arrow), maxillary (mx, bracket) and mandibular (md, arrows) branches of CN V appear dysmorphic in *LgDel* E10.5 embryos. Second row: the bifurcation (arrows) of CN VII that prefigures its division into multiple dorsal and ventral branches, including the chorda tympani, is frequently not evident in *LgDel* embryos. Third row: the sensory ganglia of CN IX/X and their immediate distal branches are frequently fused in *LgDel* embryos (compare arrows in left and right panels).

Changes in rhombomere differentiation, gene expression and patterning together with postnatal anomalies in growth, feeding and craniofacial morphogenesis in *LgDel* mice suggest that CN development, which is essential for normal oro-facial function (reviewed by Trainor and Krumlauf, 2000; Cordes, 2001), might be altered. To evaluate potential sensitivity of developing CNs to 22q11 gene dosage, we first assessed 22q11 gene expression in microdissected samples of CN ganglion (CNg) V from *LgDel* and WT embryos. 16 out of 21 22q11 genes, nearly identical to those in the hindbrain (see Fig. 4), are expressed in CNg V (Fig. 5, top panel). This substantial local CN expression of 22q11 genes, as well as E9.5 hindbrain anomalies, suggests that CN differentiation might be altered in the *LgDel* mouse at E10.5. We immunolabeled CN axons and assessed their position and growth in whole E10.5 embryos. CNs and CNgs (CN V and VII) associated with the anterior hindbrain appeared compressed, consistent with morphogenetic, gene expression and patterning changes from r4 to r1 at E9.5 (see Fig. 4). The distance between CNg V and CNg VII, whose development depends upon appropriate r1 to r4 patterning (reviewed by Cordes, 2001), was diminished (Fig. 5, middle left panel). Similarly, the position of CN V and CN VII motor roots was altered and they were less branched (Fig. 5, middle left panel, asterisks). Finally, CNg X and CNg IX were compressed, and their motor roots altered (Fig. 5, middle left panel, arrows).

Based upon these changes in CN differentiation, we evaluated expression levels of adhesion molecules and transcriptional regulators in CNg V to determine whether selected regulators of CN development diverge in WT and *LgDel* embryos. We assessed chemoattractant and chemorepulsive signaling receptors, cell surface adhesion ligands, and neurotrophin receptors. *Robo2*, one of the receptors for the Slit family of secreted adhesion molecules and which has previously been associated with trigeminal gangliogenesis (Shiau et al., 2008), as well as the *L1Cam* adhesion molecule that influences axon fasciculation and guidance, are increased in the E10.5 *LgDel* CNg V (Fig. 5, bottom left panel). The *Robo2* increase was significant (*P*≤0.05, *t*-test; *n*=8 *LgDel*, 12 WT), whereas that for *L1Cam* showed a trend (*P*≤0.06, *t*-test). Thus, diminished dosage of 22q11 genes modestly alters gene expression levels for a subset of adhesion signaling molecules in CNg V.

**Fig. 6.**
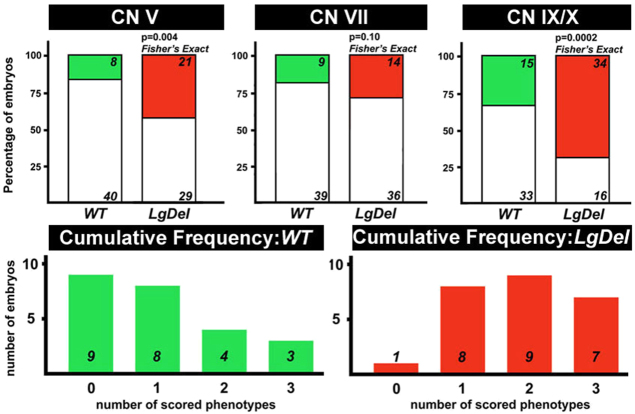
**Statistically significant changes in phenotypic frequency in developing CN V, IX and X in *LgDel* versus WT embryos.** Top panels: histograms showing the proportion of WT (green) and *LgDel* (red) embryos with each of the three phenotypes scored (CN V, VII and IX/X). Statistical significance determined using Fisher’s exact analysis. The CN V and IX/X phenotypes occur at significantly greater frequency than in the WT; the CN VII phenotype does not. Lower panels: frequency of single or multiple CN phenotypes in WT and *LgDel* embryos. Overall phenotypic frequency is substantially increased in *LgDel* mice, as is the number of individual embryos showing multiple (two or three) CN phenotypes.

These changes indicate potential divergence in *LgDel* versus WT CN differentiation, perhaps distinguishing anterior versus posterior CNs. We identified three distinct features of CN morphology that were consistently altered in a sample of 24 *LgDel* E10.5 embryos compared with 23 WT controls (Fig. 5, bottom right panels): diminished complexity and apparent length of the maxillary branch of the trigeminal nerve (CN V; Fig. 5, first row, bottom right); absence of bifurcation of the facial nerve within BA2 (CN VII; Fig. 5, second row, bottom right panel); and anomalous axon fascicles between, or actual fusion of, the glossopharyngeal and vagus sensory ganglia and nerve (CN IX and X; Fig. 5, third row, bottom right). We also examined the hypoglossal nerve (CN XII) but found variability incompatible with consistent scoring. CN V, VII, IX and X anomalies were seen occasionally in WT embryos; however, the severity and frequency was substantially increased in *LgDel* embryos. When each phenotype was scored blind by four independent observers, we found a significant increase in *LgDel* CN V and CN IX/X phenotypes (Fig. 6, top). CN V anomalies occurred in a higher percentage of *LgDel* (42%) versus WT (16%, *P*<0.005; Fisher’s exact test) embryos. CN VII branching failure also occurred at a higher frequency in the *LgDel* (28%) versus WT (18%) mice; however, this difference did not reach significance (*P*≤0.1). CN IX/X fusion was the most frequent and statistically robust phenotype (*LgDel*: 68%, WT: 31%, *P*≤0.0005). Finally, multiple phenotypes (≥2) in a single embryo were significantly more frequent in *LgDel* (Fig. 6, bottom; *P*≤0.002). Thus, diminished 22q11 gene dosage results in significant CN developmental phenotypes.

### Posterior versus anterior CN phenotypes depend upon *Tbx1* dosage

**Fig. 7.**
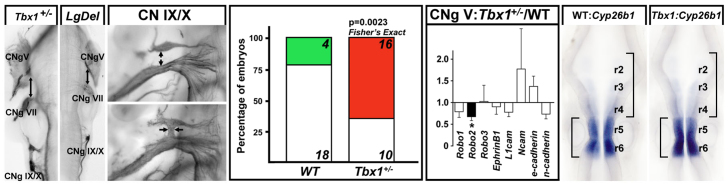
**Heterozygous *Tbx1* mutation yields a CN IX/X phenotype parallel to that in E10.5 *LgDel* embryos.** Left: the ganglia of CN IX and CN X are more frequently connected by axon fascicles or fused (arrows) in E10.5 *Tbx1*^+/−^ embryos than WT littermates. Middle left: quantitative analysis of CN phenotypes shows a statistically significant CN IX/X phenotype with the same frequency in *Tbx1*^+/−^ embryos as in *LgDel* embryos. Middle right: qPCR measurement of gene expression in the CN V ganglion of E10.5 *Tbx1*^+/−^ embryos, normalized to WT littermate control levels. *Robo2* (black bar), which increases significantly in the *LgDel* (see Fig. 5), decreases significantly in the *Tbx1*^+/−^ CN V ganglion (asterisk). Right: *Cyp26b1*, which, in WT, is expressed at high levels in r5/6, lower levels in r3/4 and barely detectable in r2, has a similar pattern of expression in r6 through r2 in *Tbx1*^+/−^ embryos. There is no noticeable expansion of r5/6 (compare brackets left) or compression of r2 to r4 (compare brackets right).

CNg IX and X fusions similar to those in *LgDel* embryos have been reported in *Tbx1^−/−^* mutants (Vitelli et al., 2002), and might reflect altered peripheral neural crest migration (Calmont et al., 2009). We did not see anterior hindbrain compression or aberrant spacing of CN V and VII in E10.5 *Tbx1*^+/−^ embryos as in the *LgDel* (Fig. 7, far left panel). We did not find the *LgDel* CN V phenotype or substantial disruption of CN VII in 13 *Tbx1*^+/−^ versus 11 WT littermates (26 and 22 individual nerves/ganglia analyzed, respectively, for each phenotype). We did find a significantly higher frequency of CN IX/X ganglia fusion in *Tbx1*^+/−^ versus WT embryos (Fig. 7; *P*≤0.05) that approximates that seen in *LgDel* (*Tbx1*^+/−^: 62% versus *LgDel*: 68%). Despite this similarity, *Tbx1*^+/−^ pups do not display the P7 weight difference seen in *LgDel* counterparts (*Tbx1*^+/−^: 4.11 g, *n*=7; WT littermates: 4.16 g, *n*=11; *P*=0.89). Expression of *L1Cam*, modestly increased in *LgDel* CNg V (with a significant trend; see Fig. 5), did not differ in *Tbx1*^+/−^ versus WT; however, *Robo2* was significantly reduced (34%; *P*<0.05; Fig. 7), opposite to the 36% increase in *LgDel* (Fig. 4). Finally, there was no substantial expression intensity difference or anterior expansion of *Cyp26b1* in E9.5 *Tbx1*^+/−^ vs WT littermate embryos hybridized concurrently (7 *Tbx1*^+/−^; 3 WT; Fig. 7, far right). Thus, posterior CN IX/X phenotypes in *LgDel* embryos reflect diminished *Tbx1* dosage – one of a small number of 22q11 genes not expressed in the developing hindbrain – whereas those associated with anterior CN V phenotypes are associated with diminished expression of additional 22q11 genes and local changes of gene expression levels or patterns, especially in the anterior hindbrain.

### Diminished RA signaling rescues anterior CN phenotypes in *LgDel* embryos

**Fig. 8.**
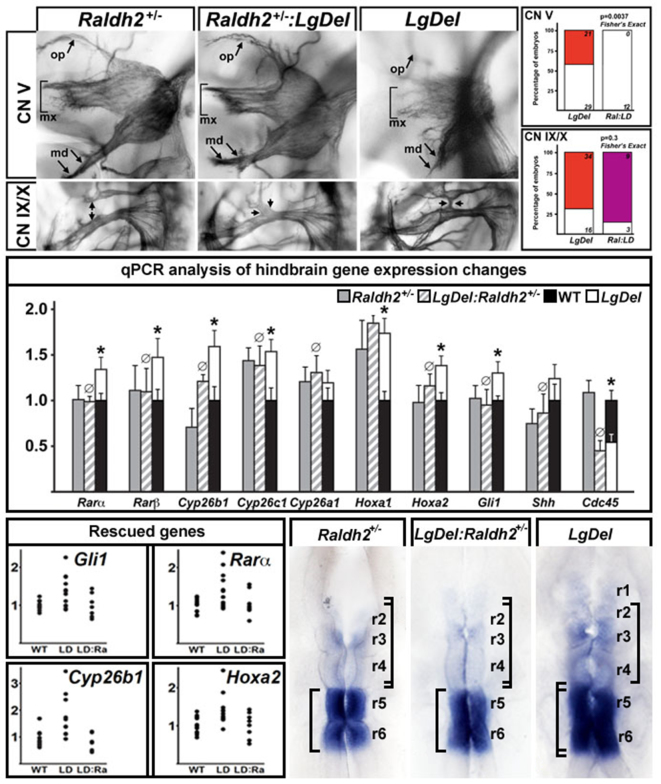
**Heterozygous inactivation of *Raldh2* rescues CN V, but not CN IX/X, phenotypes in *LgDel* embryos.** Top left: (first row) comparison of CN V differentiation in E10.5 *Raldh2*^+/−^, which resembles the WT (see Fig. 5), *Raldh2*^+/−^:*LgDel*, which also resembles the WT, and *LgDel*, which shows clear disruption of fasciculation and differentiation of all major branches of CN V. The ophthalmic (op, arrow), maxillary (mx, bracket) and mandibular (md, arrows) branches of CN V are shown. (Second row) Comparison of CN IX/X differentiation in E10.5 *Raldh2*^+/−^, *Raldh2*^+/−^:*LgDel* and *LgDel* embryos. CNs in *Raldh2*^+/−^ embryos resemble the WT, whereas *Raldh2*^+/−^:*LgDel* and *LgDel* have similar ganglion fusions (arrows) and disrupted axon trajectories. Top right: frequency of phenotypes in 12 individual CN V and CN IX/X from six E10.5 *Raldh2*^+/−^:*LgDel*, or from six *LgDel* embryos. Statistical comparisons made using Fisher’s exact analysis. Middle: A-P rhombomere selective genes, many of which are RA regulated (see Fig. 4), return to WT levels in E9.5 *LgDel:Raldh2*^+/−^ hindbrains, based upon qPCR analysis of microdissected hindbrain samples from *n*=8 *Raldh2*^+/−^; 7 *LgDel:Raldh2*^+/−^; 13 WT; and 12 *LgDel* embryos. Ø: genes for which *LgDel:Raldh2*^+/−^ levels are statistically indistinguishable from WT and *Raldh2*^+/−^ levels; *: genes for which *LgDel* levels are significantly increased over both *Raldh2*^+/−^ and WT. Bottom left: scatterplots showing the ranges of individual hindbrain expression values for four ‘rescued’ genes in WT, *LgDel* (LD) and *LgDel:Raldh2*^+/−^ (LD:Ra). The minimum and maximum values in the WT and *LgDel:Raldh2*^+/−^ are similar, and the minimum and maximum values for the *LgDel* are consistently increased. Bottom right: *Cyp26b1* patterns in the *Raldh2*^+/−^ hindbrain are similar to WT (compare to WT panels in Figs 4 and 7); those in *LgDel:Raldh2*^+/−^ hindbrain resemble the *Raldh2*^+/−^ and WT as well; in the *LgDel*, intensity increases in r6, r5, r4 and r3, the barely detectable expression in r2 is more robust, and, in this case, apparently extends into r1. Right hand brackets show expansion of r5/6 in *LgDel* but not *Raldh2*^+/−^ or *LgDel:Raldh2*^+/−^ hindbrain; left brackets show that r2-r4 are apparently compressed in the *LgDel* but not *Raldh2*^+/−^ or *LgDel:Raldh2*^+/−^ hindbrain.

Increased expression or expanded pattern of RA-regulated genes (reviewed by Glover et al., 2006) suggest disruptions of A-P patterning via enhanced RA signaling in the *LgDel* hindbrain. The distinction between CN V and CN IX/X phenotypes in *LgDel* versus *Tbx1*^+/−^ mice also suggests potentially separable mechanisms for anterior versus posterior CN disruption in the context of 22q11 deletion. Thus, we investigated whether genetic manipulation of RA signaling due to heterozygous mutation of *Raldh2*, which diminishes RA signaling in whole E10.5 embryos by 20% (Maynard et al., 2013), modifies *LgDel* anterior versus posterior CN phenotypes. In *LgDel:Raldh2*^+/−^ compound embryos, the CN V phenotype was consistently rescued (Fig. 8, top). In blind scoring of 12 CN V from 6 compound embryos, all were identified as non-phenotypic by four independent observers (Fig. 8, middle). This phenotypic frequency in *LgDel:Raldh2*^+/−^ was significantly different from that in *LgDel* (0 versus 42%; *P*≤0.004; Fisher’s exact test). In contrast, the CN IX and X phenotypic frequency was not significantly altered in *LgDel:Raldh2*^+/−^ embryos (87.5% *LgDel:Raldh2^+/−^*; 68% *LgDel; P*≤0.3; Fisher’s exact test). Apparently, diminished *Raldh2* activity rescues anterior (CN V) but not posterior (CN IX/X) *LgDel* CN phenotypes.

We next investigated whether this apparent anterior *LgDel* rescue by *Raldh2^+/−^* returns *LgDel* expression levels of RA-regulated signaling and transcription factors (see Fig. 4) toward those in WT by qPCR in microdissected hindbrain samples (r8 to r1) from E9.5 WT, *Raldh2*^+/−^, *LgDel* and *LgDel:Raldh2*^+/−^ embryos. Levels of all but one of these genes (*Hoxa1*) were statistically indistinguishable in the WT and *Raldh2*^+/−^ hindbrain (Fig. 8, middle panel). *Hoxa1* in the *Raldh2*^+/−^ and *LgDel:Raldh2*^+/−^ hindbrain remained at *LgDel* levels (increased nearly twofold over WT; Fig. 8, middle, right). Apparently, heterozygous *Raldh2* deletion, and presumed RA signaling decrement (Maynard et al., 2013), restored WT expression levels of most, but not all, RA-regulated, rhombomere-restricted genes in *LgDel:Raldh2*^+/−^ embryos. In contrast, there were no differences between genotypes for any of these genes in the E9.5 cervical and thoracic spinal cord (data not shown), indicating specificity of localized hindbrain expression changes in response to *Raldh*^+/−^. These changes are paralleled by diminished variability in individual *LgDel:Raldh2*^+/−^ gene expression toward WT ranges (Fig. 8, lower left). Finally, increased *Cyp26b1* labeling intensity in *LgDel* r3-r6 as well as anterior expansion into r2 (see Figs 4, 7) was not typically seen in the *Raldh2*^+/−^ (9/11) or *LgDel:Raldh2*^+/−^ embryos (7/11). Apparently, restoration of WT expression levels and patterns of RA-regulated, rhombomere-restricted genes accompanies *Raldh2^+/−^* rescue of CN V phenotypes.

## DISCUSSION

The *LgDel* 22q11DS mouse recapitulates several features of perinatal dysphagia in 22q11DS patients: diminished weight gain, food aspiration, and increased frequency of naso-sinus and respiratory infections. As in 22q11 patients, palate and craniofacial dysmorphogenesis accompany these changes, with varying penetrance, in *LgDel* mice. Accordingly, the *LgDel* mouse models yet another clinically significant 22q11DS phenotype: dysphagia – and its impact on growth and health. These impairments are paralleled by 22q11-dosage-dependent developmental pathology that compromises rudimentary craniofacial structures and hindbrain-derived CNs. Altered gene expression or patterning in the embryonic hindbrain and CNg, followed by anomalous development of CN V, IX and X, precede signs of dysphagia in *LgDel* mice. Posterior CN IX and X phenotypes are primarily due to heterozygous *Tbx1* deletion, consistent with previous observations in *Tbx1*^−/−^ mice. Anterior *LgDel* CN V phenotypes, however, reflect an altered balance of A-P patterning via RA-mediated changes in the *LgDel* hindbrain. Thus, disrupted development of CNs that innervate oro-facial structures required for feeding and swallowing prefigures dysphagia-related phenotypes in the *LgDel* mouse. Similar early developmental disruptions might contribute to perinatal dysphagia in 22q11DS patients.

### Dysphagia, growth, health and craniofacial morphogenesis in *LgDel* mice

The *LgDel* mouse provides a model for developmental pathogenesis of perinatal dysphagia in 22q11DS. The divergence in *LgDel* and WT weight gain, similar to that in 22q11DS children (Tarquinio et al., 2012), emerges 4 days after birth; therefore, it is unlikely to reflect prenatal growth retardation. This deficit is likely caused by diminished food intake via nursing and exacerbated by milk-aspiration-related inflammation and infection. The presence of milk protein in the nasal turbinates and lungs and accompanying inflammation and infection by P7 indicates disrupted integrity of the physical mechanism that directs food to the esophagus rather than nasopharynx and trachea. Feeding difficulties can also be exacerbated by additional chemosensory changes in the oro-facial periphery. Altered olfactory capacity (Sobin et al., 2006) or disrupted innervation of taste receptors or tongue muscles due to CN IX, X, VII, XII anomalies might compromise appetite – as seen in older children and adults with a variety of developmental disorders whose food preferences are altered (Bennetto et al., 2007; Tavassoli and Baron-Cohen, 2012). Cardiovascular or thymic anomalies might also alter feeding and nutrition. Fourth pharyngeal arch artery anomalies in *LgDel* mice (Merscher et al., 2001; Maynard et al., 2013) might secondarily constrict the esophagus or trachea, as reported in dogs with a high frequency of polymorphisms in 22q11 orthologs (Philipp et al., 2011) as well as 22q11DS patients (Phelan et al., 2011). Congenital heart disease has been associated broadly with feeding difficulties in infancy (Jadcherla et al., 2009), as has hypercalcemia due to thymic dysfunction during later life (Grieve and Dixon, 1983; Balcombe, 1999). Interaction between these diverse mechanisms might aggravate feeding and swallowing difficulties. Our data indicates that they can be studied in *LgDel* mice.

### Disrupted CN development in 22q11DS

Diminished 22q11 gene dosage compromises initial CN innervation of oro-facial and pharyngeal structures necessary for feeding and swallowing by altering hindbrain A-P differentiation. Prior to robust CN axon growth (E10.5 and later), anterior rhombomeres appear compressed, CNg and motor roots are dysmorphic, and gene expression in both the hindbrain and CNg changes. Gene expression changes in the anterior hindbrain are extensive (see below), suggesting that specification of motor as well as sensory components of CN V and VII might be compromised. In addition, neural crest cells from anterior rhombomeres contribute to craniofacial targets of CN V, VII, IX and X involved in feeding and swallowing, suggesting potentially negative synergy for oro-facial functional development. The phenotypes we see are consistent with dual disruption of CNs and targets by 22q11 deletion. In our initial assessment of genes that regulate CN axon growth, we detected expression changes in *LgDel* CNg V. *Robo2*, which is increased in *LgDel* CNg V, is associated with phenotypes that parallel those in the *LgDel* embryo (Ma and Tessier-Lavigne, 2007; Shiau et al., 2008; Shiau and Bronner-Fraser, 2009; Kubilus and Linsenmayer, 2010). In contrast, there was a slight but significant decrease in *Robo2* in *Tbx1*^+/−^ CNg V; however, CN V axon phenotypes were not detected. Apparently, diminished *Robo2* expression, in the context of heterozygous *Tbx1* mutation, does not disrupt CN V development. It remains to be determined whether enhanced *Robo2* in *LgDel* mice influences CN V development. Changes in CNg V or other ganglia and nerves might reflect altered hindbrain neural crest specification or altered peripheral target differentiation, including epibranchial placodal contributions to the developing ganglia.

### *Tbx1*, CN development and dysphagia in 22q11DS

CN IX and X phenotypes in *LgDel* embryos were primarily due to diminished dosage of *Tbx1*, a 22q11 candidate gene associated with 22q11DS cardiovascular anomalies (reviewed by Scambler, 2010) that is not expressed in the developing hindbrain. In *Tbx1* mutants, neural crest migration into the third, fourth and sixth branchial arches is altered (Calmont et al., 2009), including that of cells that will contribute to CN IX and X ganglia as well as cells that provide a substrate for CN differentiation and axon growth. CN IX and X ganglia fusions occur at similar frequency in *Tbx1*^+/−^ and *LgDel* embryos, suggesting that diminished *Tbx1* dosage is probably responsible for this anomaly. This *Tbx1*^+/−^ phenotype is consistent with an earlier report of CNg IX/X fusion in *Tbx1^−/−^* null embryos (Vitelli et al., 2002). CN IX and X ganglia fusion likely reflects disruptions of neural crest migration, pharyngeal placode integrity, and subsequent differentiation in BA3, 4 and 6 (Raft et al., 2004; Arnold et al., 2006), perhaps exacerbated by dysmorphogenesis of cardiac and visceral targets. These defects alone, however, might not be sufficient to disrupt feeding and swallowing. We did not see diminished P7 weight in *Tbx1*^+/−^ pups similar to that in *LgDel* pups. Furthermore, there are divergent changes in gene expression between the two genotypes. Thus, our results define a circumscribed role for diminished dosage of *Tbx1* in a spectrum of phenotypes that together might account for dysphagia in 22q11DS.

### 22q11 deletion beyond *Tbx1* disrupts anterior hindbrain patterning

22q11 deletion, beyond heterozygous *Tbx1* mutation, disrupts the initial anterior hindbrain and subsequent CN patterning – especially that of CN V. The changes appear focused on anterior-most rhombomeres, r2–r3. The morphogenetic compression of the hindbrain at E9.5 and E10.5 diminishes this territory and alters the relative positions of CN V and VII. Deformations of the neuroepithelium in this region could reflect altered cell proliferation, enhanced cell death, or disrupted initial delamination and migration of the neural crest from aberrant rhombomeres. Quantitative expression changes target genes that define A-P hindbrain boundaries or anterior rhombomeres themselves. *Cyp26b1*, which shows the greatest expression level change, also expands anteriorly, with anomalous increased expression in r2, r3 and r4. Apparently, diminished 22q11 gene expression modifies the molecular and cellular identities of rhombomeres that give rise to CN V. RA regulates most of the genes selected for quantitative as well as patterning analysis – including *Cyp26b1* (Abu-Abed et al., 1998; Okano et al., 2012). Changes in expression level or pattern are consistent with increased anterior RA signaling in the *LgDel* hindbrain. The anterior expansion of *Cyp26b1* provides visual reinforcement of the qPCR analysis, which showed enhanced expression levels of a broader set of RA-regulated genes in the microdissected E9.5 *LgDel* hindbrain. Together, these data solidly support the hypothesis that diminished 22q11 gene dosage disrupts development of CN V and, perhaps, CN VII as well, via increased expression and/or anterior expansion of RA-regulated genes that distinguish anterior versus posterior rhombomeres.

Selective rescue of *LgDel* CN V phenotypes by heterozygous mutation of *Raldh2* (*Raldh2*^+/−^), which lowers RA signaling in the whole embryo by 20% (Maynard et al., 2013), establishes RA-mediated disruption of CN development as a potential contributor to dysphagia pathogenesis. Two clear conclusions emerge: first, *LgDel* anterior CN differentiation is likely to be sensitive to RA levels, whereas posterior CNs are not; second, *Tbx1* disrupts posterior CN IX/X gangliogenesis or axon growth independent of RA signaling. In contrast, RA signaling is disrupted in the heart, thymus and otic vesicle in *Tbx1* mutants (Roberts et al., 2006; Braunstein et al., 2009; Monks and Morrow, 2012), and *Raldh2*^+/−^ can rescue the thymic phenotype (Guris et al., 2006). The reduction of RA levels by *Raldh2*^+/−^ restored the expression of RA-regulated genes in the *LgDel* hindbrain to WT levels, and the return of one of these genes, *Cyp26b1*, to the WT pattern further defines a mechanism by which 22q11 deletion, exclusive of *Tbx1*, disrupts RA-regulated anterior hindbrain patterning, leading to altered CN V development. This anterior disruption, together with *Tbx1*-mediated posterior CN IX and X disruption, likely compromises the CN innervation necessary for optimal feeding and swallowing. Thus, dysphagia-associated phenotypes in *LgDel* pups reflect 22q11 contiguous genes that disrupt mechanisms of craniofacial and CN development. Specific consequences for feeding and swallowing of these aberrant mechanisms, and their amelioration by genetic or pharmacological strategies involving RA or related signaling pathways, remain to be determined.

## MATERIALS AND METHODS

### Animals

The George Washington (GW) University Animal Research Facility maintained colonies of WT C57/BL6 (Charles River Laboratories), *LgDel* (Merscher et al., 2001), *Raldh2*^+/−^ (Mic et al., 2002) and *Tbx1*^+/−^ (Jerome and Papaioannou, 2001) mice. Mutant lines were backcrossed for at least ten generations to the C57/BL6 background to generate breeding stock for these experiments. The *LgDel* mutation (heterozygous deletion on mmchr. 16 from *Idd* to *Hira*) and *Tbx1* mutations were transmitted paternally; *Raldh2*^+/−^ was transmitted maternally. All procedures were reviewed and approved by the GW Institutional Animal Care and Use Committee (IACUC).

### Animal measurements

Individual *LgDel* and WT littermate mouse pups from seven litters were identified with unique labels at postnatal day 1 (P1), and weighed each morning through P7, and then again at P14, P21, P28 and P30. All weights were collected blind to genotype. These mice were sacrificed at day P30 by CO_2_ asphyxiation, and genotyped as described previously (Maynard et al., 2003; Maynard et al., 2013). In addition, skulls were gross dissected and digested in a solution of SNET (10 mM Tris pH 8.0, 0.1 M EDTA, 0.5% SDS) and proteinase K (New England Biolabs) at 60°C for 3 days. Right and left lower mandibles were cleaned and imaged in a standard orientation on a Leica Wild M420 photomacroscope. Mandibular landmarks were established (Richtsmeier et al., 2000) and landmark-to-landmark distances were measured using ImageJ software (NIH 2012).

### Histological analysis

After CO_2_ euthanasia, heads and lungs of P7 *LgDel*, WT and *Tbx1*^+/−^ pups were collected and fixed in 4% paraformaldehyde (PFA) at 4°C. The heads were decalcified in 0.1 M EDTA in 0.1 M phosphate buffer. Heads and lungs were cryoprotected and cryo-embedded, and 10 μm serial sections were prepared and stained with hematoxylin and eosin (H&E) or periodic acid-Schiff (PAS) reagents or immunolabeled with milk protein antibodies (Biorbyt) at 1:1000 as well as anti-CD64 (neutrophil marker) at 1:200 (Santa Cruz Biotechnology). For specificity, milk antibody was pre-adsorbed to an acetone extract of E16 mouse embryos and adult mouse brain, liver and lung. Alexa-Fluor-488 and -546 species-specific secondary antibodies were used for detection. Sections were imaged using a Leica DM 6000B microscope.

### RNA isolation, cDNA synthesis and qPCR

Hindbrains were dissected from E9.5 *LgDel* and WT littermates. Maxillary [branchial arch (BA)1a] and mandibular/hyoid (BA1b/BA2) processes, as well as CNg V, were dissected from E10.5 *LgDel* and WT littermates or E10.5 *Tbx1*^+/−^ and WT littermates. RNA was prepared as described previously (Maynard et al., 2003). qPCR was performed using a Bio-Rad CFX384 Real-Time PCR detection system. Gene-specific primers, when possible, spanned genomic intron-exon boundaries and generated amplicons between 250 and 350 bp, and were validated by melt-curve analysis (qPCR Primers, Tables 1 and 2). Expression in *LgDel* or *Tbx1*^+/−^ samples is displayed as the fraction of expression in the WT littermate cohort. Mean expression values between genotypes were compared using a *t*-test (*P*≤0.05).

**Table 1.**
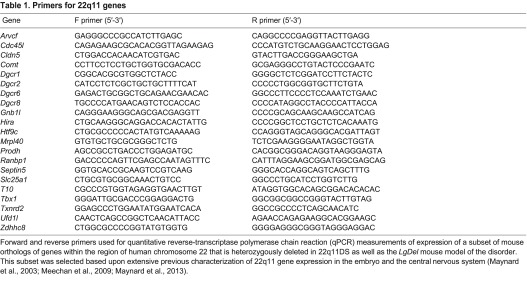
Primers for 22q11 genes

Forward and reverse primers used for quantitative reverse-transcriptase polymerase chain reaction (qPCR) measurements of expression of a subset of mouse orthologs of genes within the region of human chromosome 22 that is heterozygously deleted in 22q11DS as well as the *LgDel* mouse model of the disorder. This subset was selected based upon extensive previous characterization of 22q11 gene expression in the embryo and the central nervous system (Maynard et al., 2003; Meechan et al., 2009; Maynard et al., 2013).

**Table 2.**
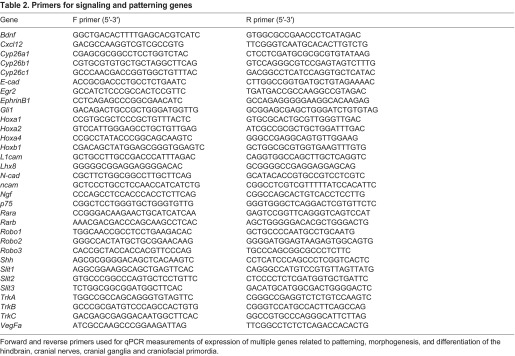
Primers for signaling and patterning genes

Forward and reverse primers used for qPCR measurements of expression of multiple genes related to patterning, morphogenesis, and differentiation of the hindbrain, cranial nerves, cranial ganglia and craniofacial primordia.

### Whole-mount immunohistochemistry

E10.5 embryos were fixed in 4% PFA at 4°C and dehydrated through a graded methanol/PBS series. Embryos were incubated in 5:1 methanol:H_2_O_2_ for 30 minutes to quench endogenous peroxidases, rehydrated and incubated for 1 hour in PBS with 0.2% BSA and 0.1% Triton X-100 (PBS-T), 10% normal goat serum (NGS), 1% Boehringer Mannheim Blocking Reagent (BMB) for 1 hour. For rhombomere analysis, rabbit polyclonal anti-Hoxb1 (r4-specific label; Covance, 1:400) and mouse monoclonal anti-En1 (r1/cerebellum/mesencephalon-specific label; Developmental Studies Hybridoma Bank; 1:400) was used and, for CNs, mouse monoclonal anti-165kDa neurofilament protein (2H3, Developmental Studies Hybridoma Bank; 1:1000) was used. Embryos were incubated in primary antibodies at 4°C for 3 days. Embryos were then washed extensively in PBST and incubated overnight in a 1:500 dilution of HRP-conjugated goat anti-rabbit and HRP-conjugated goat anti-mouse antibodies (GE Healthcare Life Sciences) for Hoxb1 and En1 or HRP-conjugated goat anti-mouse secondary antibody (GE Healthcare Life Sciences) for neurofilament/2H3. Following DAB/NiCl_2_ visualization of HRP, embryos were dehydrated, cleared with benzyl alcohol:benzyl benzoate (BABB) and imaged using a Leica Wild M420 photomacroscope.

### *In situ* hybridization

A 503-bp fragment of *Cyp26b1* (Accession number NM175475), chosen for low homology to other Cyp26 family members, was subcloned into a modified Bluescript vector, and digoxigenin-labeled probes (Roche) were made using T3 and T7 RNA polymerase (Promega). *In situ* hybridization on whole E9.5 embryos was performed as described previously (Maynard et al., 2002). Hybridized embryos were cleared in glycerol and photographed after microdissection. Expression changes described in the text were based upon independent assessments by two observers. All comparisons were done in groups of embryos hybridized concurrently.

### Assessment of phenotypes

Images of whole-mount labeled CNs and CNg were evaluated, blind to genotype, by four independent observers. Scoring criteria were as follows: for CN V, a score of 0 was assigned if there was a well-developed ganglion with clear ophthalmic, maxillary and mandibular divisions and dense axon fascicles growing toward the periphery. A score of 1 was assigned if the trigeminal ganglion was smaller in overall size, or if outgrowing fibers were short or sparse. For CN VII, a score of 0 was given if fibers of the main branch bifurcated to grow toward both BA1 (the chorda tympani branch) and BA2 (facial nerve); a score of 1 was assigned if there was no bifurcation and the fibers grew toward only BA1 or BA2. For CN IX–X, a score of 0 was assigned if there was no contact between CN IX and X. A score of 1 was given if ectopic fibers were observed growing between CN IX and X or if the ganglia were fused. These scores from four independent observers were then tallied and the mean phenotypic scores determined. A mean score of 0, 0.25 or 0.5 was reassigned a score of 0; a mean score of 0.75 or 1 was given a score of 1. Fisher’s exact test was used to compare phenotypes in groups with distinct genotypes.

## References

[b1-0070245] Abu-Abed S. S. Beckett B. R. Chiba H. Chithalen J. V. Jones G. Metzger D. Chambon P. Petkovich M. (1998). Mouse P450RAI (CYP26) expression and retinoic acid-inducible retinoic acid metabolism in F9 cells are regulated by retinoic acid receptor gamma and retinoid X receptor alpha. J. Biol. Chem. 273, 2409–2415 9442090 10.1074/jbc.273.4.2409

[b2-0070245] Ang K. K. McRitchie R. J. Minson J. B. Llewellyn-Smith I. J. Pilowsky P. M. Chalmers J. P. Arnolda L. F. (1999). Activation of spinal opioid receptors contributes to hypotension after hemorrhage in conscious rats. Am. J. Physiol. 276, H1552–H1558 10330238 10.1152/ajpheart.1999.276.5.H1552

[b3-0070245] Arnold J. S. Werling U. Braunstein E. M. Liao J. Nowotschin S. Edelmann W. Hebert J. M. Morrow B. E. (2006). Inactivation of Tbx1 in the pharyngeal endoderm results in 22q11DS malformations. Development 133, 977–987 16452092 10.1242/dev.02264

[b4-0070245] Balcombe N. R. (1999). Dysphagia and hypercalcaemia. Postgrad. Med. J. 75, 373–374 10435181 10.1136/pgmj.75.884.373 PMC1741245

[b5-0070245] Barrow J. R. Stadler H. S. Capecchi M. R. (2000). Roles of Hoxa1 and Hoxa2 in patterning the early hindbrain of the mouse. Development 127, 933–944 10662633 10.1242/dev.127.5.933

[b6-0070245] Bennetto L. Kuschner E. S. Hyman S. L. (2007). Olfaction and taste processing in autism. Biol. Psychiatry 62, 1015–1021 17572391 10.1016/j.biopsych.2007.04.019 PMC2063511

[b7-0070245] Braunstein E. M. Monks D. C. Aggarwal V. S. Arnold J. S. Morrow B. E. (2009). Tbx1 and Brn4 regulate retinoic acid metabolic genes during cochlear morphogenesis. BMC Dev. Biol. 9, 31 19476657 10.1186/1471-213X-9-31 PMC2700094

[b8-0070245] Calmont A. Ivins S. Van Bueren K. L. Papangeli I. Kyriakopoulou V. Andrews W. D. Martin J. F. Moon A. M. Illingworth E. A. Basson M. A. (2009). Tbx1 controls cardiac neural crest cell migration during arch artery development by regulating Gbx2 expression in the pharyngeal ectoderm. Development 136, 3173–3183 19700621 10.1242/dev.028902 PMC2730371

[b9-0070245] Cayé-Thomasen P. Tos M. (2003). Eustachian tube goblet cell density during and after acute otitis media caused by Streptococcus pneumoniae: a morphometric analysis. Otol. Neurotol. 24, 365–370 12806285 10.1097/00129492-200305000-00003

[b10-0070245] Christensen J. R. (1989). Developmental approach to pediatric neurogenic dysphagia. Dysphagia 3, 131–134 2639770 10.1007/BF02407131

[b11-0070245] Cordes S. P. (2001). Molecular genetics of cranial nerve development in mouse. Nat. Rev. Neurosci. 2, 611–623 11533729 10.1038/35090039

[b12-0070245] Eicher P. S. McDonald-Mcginn D. M. Fox C. A. Driscoll D. A. Emanuel B. S. Zackai E. H. (2000). Dysphagia in children with a 22q11.2 deletion: unusual pattern found on modified barium swallow. J. Pediatr. 137, 158–164 10931405 10.1067/mpd.2000.105356

[b13-0070245] Emanuel B. S. McDonald-McGinn D. Saitta S. C. Zackai E. H. (2001). The 22q11.2 deletion syndrome. Adv. Pediatr. 48, 39–73 11480765

[b14-0070245] Friedman M. A. Miletta N. Roe C. Wang D. Morrow B. E. Kates W. R. Higgins A. M. Shprintzen R. J. (2011). Cleft palate, retrognathia and congenital heart disease in velo-cardio-facial syndrome: a phenotype correlation study. Int. J. Pediatr. Otorhinolaryngol. 75, 1167–1172 21763005 10.1016/j.ijporl.2011.06.013 PMC3162093

[b15-0070245] Fukui N. Amano A. Akiyama S. Daikoku H. Wakisaka S. Morisaki I. (2000). Oral findings in DiGeorge syndrome: clinical features and histologic study of primary teeth. Oral Surg. Oral Med. Oral Pathol. Oral Radiol. Endod. 89, 208–215 10673658 10.1067/moe.2000.103884

[b16-0070245] Glover J. C. Renaud J. S. Rijli F. M. (2006). Retinoic acid and hindbrain patterning. J. Neurobiol. 66, 705–725 16688767 10.1002/neu.20272

[b17-0070245] Grieve R. J. Dixon P. F. (1983). Dysphagia: a further symptom of hypercalcaemia? Br. Med. J. 286, 1935–1936 6407643 10.1136/bmj.286.6382.1935 PMC1548315

[b18-0070245] Guris D. L. Duester G. Papaioannou V. E. Imamoto A. (2006). Dose-dependent interaction of Tbx1 and Crkl and locally aberrant RA signaling in a model of del22q11 syndrome. Dev. Cell 10, 81–92 16399080 10.1016/j.devcel.2005.12.002

[b19-0070245] Hernandez R. E. Putzke A. P. Myers J. P. Margaretha L. Moens C. B. (2007). Cyp26 enzymes generate the retinoic acid response pattern necessary for hindbrain development. Development 134, 177–187 17164423 10.1242/dev.02706 PMC1765950

[b20-0070245] Hopkin R. J. Schorry E. K. Bofinger M. Saal H. M. (2000). Increased need for medical interventions in infants with velocardiofacial (deletion 22q11) syndrome. J. Pediatr. 137, 247–249 10931419 10.1067/mpd.2000.108272

[b21-0070245] Huang R. Y. Shapiro N. L. (2000). Structural airway anomalies in patients with DiGeorge syndrome: a current review. Am. J. Otolaryngol. 21, 326–330 11032298 10.1053/ajot.2000.16166

[b22-0070245] Jadcherla S. R. Vijayapal A. S. Leuthner S. (2009). Feeding abilities in neonates with congenital heart disease: a retrospective study. J. Perinatol. 29, 112–118 18818664 10.1038/jp.2008.136 PMC3799769

[b23-0070245] Jawad A. F. McDonald-Mcginn D. M. Zackai E. Sullivan K. E. (2001). Immunologic features of chromosome 22q11.2 deletion syndrome (DiGeorge syndrome/velocardiofacial syndrome). J. Pediatr. 139, 715–723 11713452 10.1067/mpd.2001.118534

[b24-0070245] Jerome L. A. Papaioannou V. E. (2001). DiGeorge syndrome phenotype in mice mutant for the T-box gene, Tbx1. Nat. Genet. 27, 286–291 11242110 10.1038/85845

[b25-0070245] Kelly M. M. (2006). Primary care issues for the healthy premature infant. J. Pediatr. Health Care 20, 293–299 16962433 10.1016/j.pedhc.2006.01.002

[b26-0070245] Kobrynski L. J. Sullivan K. E. (2007). Velocardiofacial syndrome, DiGeorge syndrome: the chromosome 22q11.2 deletion syndromes. Lancet 370, 1443–1452 17950858 10.1016/S0140-6736(07)61601-8

[b27-0070245] Kubilus J. K. Linsenmayer T. F. (2010). Developmental guidance of embryonic corneal innervation: roles of Semaphorin3A and Slit2. Dev. Biol. 344, 172–184 20471970 10.1016/j.ydbio.2010.04.032 PMC4283142

[b28-0070245] Lefton-Greif M. A. (2008). Pediatric dysphagia. Phys. Med. Rehabil. Clin. N. Am. 19, 837–851, ix. (ix.) 18940644 10.1016/j.pmr.2008.05.007

[b29-0070245] Lima K. Følling I. Eiklid K. L. Natvig S. Abrahamsen T. G. (2010). Age-dependent clinical problems in a Norwegian national survey of patients with the 22q11.2 deletion syndrome. Eur. J. Pediatr. 169, 983–989 20186429 10.1007/s00431-010-1161-3

[b30-0070245] Lundy D. S. Smith C. Colangelo L. Sullivan P. A. Logemann J. A. Lazarus C. L. Newman L. A. Murry T. Lombard L. Gaziano J. (1999). Aspiration: cause and implications. Otolaryngol. Head Neck Surg. 120, 474–478 10187936 10.1053/hn.1999.v120.a91765

[b31-0070245] Ma L. Tessier-Lavigne M. (2007). Dual branch-promoting and branch-repelling actions of Slit/Robo signaling on peripheral and central branches of developing sensory axons. J. Neurosci. 27, 6843–6851 17581972 10.1523/JNEUROSCI.1479-07.2007 PMC6672698

[b32-0070245] Marom T. Roth Y. Goldfarb A. Cinamon U. (2012). Head and neck manifestations of 22q11.2 deletion syndromes. Eur. Arch. Otorhinolaryngol. 269, 381–387 21861138 10.1007/s00405-011-1745-1

[b33-0070245] Marshall H. Nonchev S. Sham M. H. Muchamore I. Lumsden A. Krumlauf R. (1992). Retinoic acid alters hindbrain Hox code and induces transformation of rhombomeres 2/3 into a 4/5 identity. Nature 360, 737–741 1361214 10.1038/360737a0

[b34-0070245] Maynard T. M. Haskell G. T. Bhasin N. Lee J. M. Gassman A. A. Lieberman J. A. LaMantia A. S. (2002). RanBP1, a velocardiofacial/DiGeorge syndrome candidate gene, is expressed at sites of mesenchymal/epithelial induction. Mech. Dev. 111, 177–180 11804793 10.1016/s0925-4773(01)00616-5

[b35-0070245] Maynard T. M. Haskell G. T. Peters A. Z. Sikich L. Lieberman J. A. LaMantia A. S. (2003). A comprehensive analysis of 22q11 gene expression in the developing and adult brain. Proc. Natl. Acad. Sci. USA 100, 14433–14438 14614146 10.1073/pnas.2235651100 PMC283609

[b36-0070245] Maynard T. M. Gopalakrishna D. Meechan D. W. Paronett E. M. Newbern J. M. LaMantia A. S. (2013). 22q11 Gene dosage establishes an adaptive range for sonic hedgehog and retinoic acid signaling during early development. Hum. Mol. Genet. 22, 300–312 23077214 10.1093/hmg/dds429 PMC3526161

[b37-0070245] Meechan D. W. Tucker E. S. Maynard T. M. LaMantia A. S. (2009). Diminished dosage of 22q11 genes disrupts neurogenesis and cortical development in a mouse model of 22q11 deletion/DiGeorge syndrome. Proc. Natl. Acad. Sci. USA 106, 16434–16445 19805316 10.1073/pnas.0905696106 PMC2752572

[b38-0070245] Merscher S. Funke B. Epstein J. A. Heyer J. Puech A. Lu M. M. Xavier R. J. Demay M. B. Russell R. G. Factor S. (2001). TBX1 is responsible for cardiovascular defects in velo-cardio-facial/DiGeorge syndrome. Cell 104, 619–629 11239417 10.1016/s0092-8674(01)00247-1

[b39-0070245] Mic F. A. Haselbeck R. J. Cuenca A. E. Duester G. (2002). Novel retinoic acid generating activities in the neural tube and heart identified by conditional rescue of Raldh2 null mutant mice. Development 129, 2271–2282 11959834 10.1242/dev.129.9.2271 PMC2833017

[b40-0070245] Monks D. C. Morrow B. E. (2012). Identification of putative retinoic acid target genes downstream of mesenchymal Tbx1 during inner ear development. Dev. Dyn. 241, 563–573 22275070 10.1002/dvdy.23731 PMC3282991

[b41-0070245] Okano J. Sakai Y. Shiota K. (2008). Retinoic acid down-regulates Tbx1 expression and induces abnormal differentiation of tongue muscles in fetal mice. Dev. Dyn. 237, 3059–3070 18816858 10.1002/dvdy.21715

[b42-0070245] Okano J. Kimura W. Papaionnou V. E. Miura N. Yamada G. Shiota K. Sakai Y. (2012). The regulation of endogenous retinoic acid level through CYP26B1 is required for elevation of palatal shelves. Dev. Dyn. 241, 1744–1756 22972661 10.1002/dvdy.23862

[b43-0070245] Ousley O. Rockers K. Dell M. L. Coleman K. Cubells J. F. (2007). A review of neurocognitive and behavioral profiles associated with 22q11 deletion syndrome: implications for clinical evaluation and treatment. Curr. Psychiatry Rep. 9, 148–158 17389127 10.1007/s11920-007-0085-8

[b44-0070245] Phelan E. Ryan S. Rowley H. (2011). Vascular rings and slings: interesting vascular anomalies. J. Laryngol. Otol. 125, 1158–1163 21854690 10.1017/S0022215111001605

[b45-0070245] Philipp U. Menzel J. Distl O. (2011). A rare form of persistent right aorta arch in linkage disequilibrium with the DiGeorge critical region on CFA26 in German Pinschers. J. Hered. 102 Suppl. 1, S68–S73 21846749 10.1093/jhered/esr053

[b46-0070245] Raft S. Nowotschin S. Liao J. Morrow B. E. (2004). Suppression of neural fate and control of inner ear morphogenesis by Tbx1. Development 131, 1801–1812 15084464 10.1242/dev.01067

[b47-0070245] Reijntjes S. Gale E. Maden M. (2003). Expression of the retinoic acid catabolising enzyme CYP26B1 in the chick embryo and its regulation by retinoic acid. Gene Expr. Patterns 3, 621–627 12971996 10.1016/s1567-133x(03)00112-1

[b48-0070245] Reilly S. M. Skuse D. H. Wolke D. Stevenson J. (1999). Oral-motor dysfunction in children who fail to thrive: organic or non-organic? Dev. Med. Child Neurol. 41, 115–122 10075097 10.1017/s0012162299000225

[b49-0070245] Richtsmeier J. T. Baxter L. L. Reeves R. H. (2000). Parallels of craniofacial maldevelopment in Down syndrome and Ts65Dn mice. Dev. Dyn. 217, 137–145 10706138 10.1002/(SICI)1097-0177(200002)217:2<137::AID-DVDY1>3.0.CO;2-N

[b50-0070245] Roberts C. Ivins S. Cook A. C. Baldini A. Scambler P. J. (2006). Cyp26 genes a1, b1 and c1 are down-regulated in Tbx1 null mice and inhibition of Cyp26 enzyme function produces a phenocopy of DiGeorge Syndrome in the chick. Hum. Mol. Genet. 15, 3394–3410 17047027 10.1093/hmg/ddl416

[b51-0070245] Rommel N. Davidson G. Cain T. Hebbard G. Omari T. (2008). Videomanometric evaluation of pharyngo-oesophageal dysmotility in children with velocardiofacial syndrome. J. Pediatr. Gastroenterol. Nutr. 46, 87–91 18162840 10.1097/01.mpg.0000304460.07423.68

[b52-0070245] Ruda J. M. Krakovitz P. Rose A. S. (2012). A review of the evaluation and management of velopharyngeal insufficiency in children. Otolaryngol. Clin. North Am. 45, 653–669, viii. (viii.) 22588042 10.1016/j.otc.2012.03.005

[b53-0070245] Scambler P. J. (2010). 22q11 deletion syndrome: a role for TBX1 in pharyngeal and cardiovascular development. Pediatr. Cardiol. 31, 378–390 20054531 10.1007/s00246-009-9613-0

[b54-0070245] Schwarz S. M. Corredor J. Fisher-Medina J. Cohen J. Rabinowitz S. (2001). Diagnosis and treatment of feeding disorders in children with developmental disabilities. Pediatrics 108, 671–676 11533334 10.1542/peds.108.3.671

[b55-0070245] Shiau C. E. Bronner-Fraser M. (2009). N-cadherin acts in concert with Slit1-Robo2 signaling in regulating aggregation of placode-derived cranial sensory neurons. Development 136, 4155–4164 19934013 10.1242/dev.034355 PMC2781051

[b56-0070245] Shiau C. E. Lwigale P. Y. Das R. M. Wilson S. A. Bronner-Fraser M. (2008). Robo2-Slit1 dependent cell-cell interactions mediate assembly of the trigeminal ganglion. Nat. Neurosci. 11, 269–276 18278043 10.1038/nn2051

[b57-0070245] Sobin C. Kiley-Brabeck K. Dale K. Monk S. H. Khuri J. Karayiorgou M. (2006). Olfactory disorder in children with 22q11 deletion syndrome. Pediatrics 118, e697–e703 16908619 10.1542/peds.2005-3114 PMC2753868

[b58-0070245] Tahayato A. Dollé P. Petkovich M. (2003). Cyp26C1 encodes a novel retinoic acid-metabolizing enzyme expressed in the hindbrain, inner ear, first branchial arch and tooth buds during murine development. Gene Expr. Patterns 3, 449–454 12915310 10.1016/s1567-133x(03)00066-8

[b59-0070245] Tarquinio D. C. Jones M. C. Jones K. L. Bird L. M. (2012). Growth charts for 22q11 deletion syndrome. Am. J. Med. Genet. A. 158A, 2672–2681 22887711 10.1002/ajmg.a.35485

[b60-0070245] Tavassoli T. Baron-Cohen S. (2012). Taste identification in adults with autism spectrum conditions. J. Autism Dev. Disord. 42, 1419–1424 22006402 10.1007/s10803-011-1377-8

[b61-0070245] Trainor P. A. Krumlauf R. (2000). Patterning the cranial neural crest: hindbrain segmentation and Hox gene plasticity. Nat. Rev. Neurosci. 1, 116–124 11252774 10.1038/35039056

[b62-0070245] Trinick R. Johnston N. Dalzell A. M. McNamara P. S. (2012). Reflux aspiration in children with neurodisability – a significant problem, but can we measure it? J. Pediatr. Surg. 47, 291–298 22325378 10.1016/j.jpedsurg.2011.11.019

[b63-0070245] Vitelli F. Morishima M. Taddei I. Lindsay E. A. Baldini A. (2002). Tbx1 mutation causes multiple cardiovascular defects and disrupts neural crest and cranial nerve migratory pathways. Hum. Mol. Genet. 11, 915–922 11971873 10.1093/hmg/11.8.915

[b64-0070245] Zori R. T. Boyar F. Z. Williams W. N. Gray B. A. Bent-Williams A. Stalker H. J. Rimer L. A. Nackashi J. A. Driscoll D. J. Rasmussen S. A. (1998). Prevalence of 22q11 region deletions in patients with velopharyngeal insufficiency. Am. J. Med. Genet. 77, 8–11 9557885

